# Development and Optimization of Ferrochrome Production Using Pre-Reduced Chromite Pellets

**DOI:** 10.3390/ma19112225

**Published:** 2026-05-25

**Authors:** Yerbolat Makhambetov, Ainash Akmanova, Armat Zhakan, Aibar Myrzagaliyev, Dastan Aubakirov, Zhadiger Sadyk, Zhalgas Saulebek

**Affiliations:** 1 Chemical-Metallurgical Institute Named After Zh. Abishev, Karaganda 100030, Kazakhstan; makhambetovyerbolat@gmail.com (Y.M.); sadzhad03@gmail.com (Z.S.); zhaga1998@gmail.com (Z.S.); 2 Department of Nanotechnology and Metallurgy, Abylkas Saginov Karaganda Technical University, Karaganda 100027, Kazakhstan; aibar.myrzagaliyev@erg.kz (A.M.); d.aubakirov@ktu.edu.kz (D.A.); 3 ERG Research and Engineering Center LLP, Astana 010000, Kazakhstan

**Keywords:** high-carbon ferrochrome, chromite pellets, pre-reduction, degree of metallization, semi-coke, rotary kiln, energy efficiency

## Abstract

This study investigates the production of high-carbon ferrochrome (HCFeCr) using pre-reduced chromite pellets. Chromite ore from the Kempirsai deposit, semicoke as a reducing agent, and activated bentonite as a binder were used for pellet preparation. Pellets with a size of 12–14 mm were produced and subjected to reduction roasting at 1400 °C for 1–3 h. The results showed that increasing the roasting time promoted chromite reduction and increased the chromium metallization degree. After 3 h of roasting, the chromium metallization degree reached 43.93%. SEM analysis confirmed the formation of metallized chromium-containing phases and a porous structure favorable for subsequent smelting. Smelting experiments were carried out in a 0.1 MVA ore-thermal furnace using pre-reduced pellets. Stable furnace operation, satisfactory slag fluidity, and effective separation of metal and slag were observed. The obtained high-carbon ferrochrome contained 68.92 wt.% Cr, 1.54 wt.% Si, and 7.11 wt.% C. Chromium recovery into the alloy reached 92.17%, while the slag contained 2.14 wt.% Cr_2_O_3_. The specific electric energy consumption during experimental smelting was 4648.1 kWh/t of ferrochrome. Recalculation to industrial conditions showed an expected energy consumption of 3132.76 kWh/t, confirming the potential of pre-reduced chromite pellets for energy-efficient ferrochrome production.

## 1. Introduction

High-carbon ferrochrome (HCFeCr) is the primary chromium-containing alloy used in stainless steel production, ensuring stable demand and driving the need for improvements in production efficiency. The majority of ferrochrome is produced in submerged arc furnaces (SAF), where complex physicochemical processes of chromite reduction and slag–metal interaction occur [1,2,3,4]. This process is characterized by high energy consumption, with specific electricity consumption largely determined by charge composition, raw material characteristics, and furnace operating conditions [5,6].

In general, the production of HCFeCr in SAF is one of the most energy-intensive pyrometallurgical processes, with electricity consumption typically ranging from 2400 to 4700 kWh per ton of alloy [7,8]. Conventional open furnaces usually operate within 4000–4300 kWh/t, while the use of closed furnaces with pelletized and preheated feed reduces consumption to 3100–3400 kWh/t. The lowest energy demand is achieved when pre-reduction is combined with smelting in closed furnaces, where electricity consumption can decrease to approximately 2400 kWh/t (excluding the energy required for the pre-reduction stage). Under conditions of increasing energy costs and decarbonization requirements, reducing energy consumption remains a critical challenge for ferrochrome production [1].

In the context of Kazakhstan, which possesses significant chromite reserves and a well-developed ferroalloy industry, the issue of reducing specific energy consumption in HCFeCr production remains unresolved. This is associated with the mineralogical characteristics of raw materials, the use of fine fractions, and the increasing involvement of technogenic materials and intermediate products. At the same time, conventional technologies are accompanied by considerable chromium losses to slag and high energy consumption [9,10].

One of the most promising approaches to improving ferrochrome production efficiency is the preliminary preparation of chromite raw materials, including agglomeration of fine particles and their partial solid-state reduction prior to smelting. This approach allows part of the endothermic reduction reactions to occur outside the electric arc zone, thereby reducing furnace load and specific energy consumption [11,12].

In industrial practice, this concept is implemented through pelletization followed by thermal treatment. The efficiency of agglomerated materials is determined by their physicochemical properties. The mineralogical composition of raw materials significantly affects pellet strength, porosity, and thermal stability [13]. The selection of carbonaceous reductants influences reduction kinetics, degree of metallization, and phase composition [10], while pre-treatment affects mineral structure and reducibility [14]. Recent studies have demonstrated the significant influence of reduction mechanisms, phase transformations, and microstructural evolution during the reduction roasting of oxide pellets under reducing atmospheres, which strongly affect the metallization behavior and final properties of the reduced material [15].

The development of pre-reduction technologies, including processes such as the Höganäs method, enables a high degree of metallization prior to smelting [16]. Practical studies have demonstrated that the use of pre-reduced materials improves chromium recovery and overall process performance [12,17]. Additional benefits can be achieved through the utilization of technogenic materials and intermediate products in the charge [18,19].

Despite these advances, the relationships between charge composition, reduction roasting parameters, and the properties of metallized chromite pellets remain insufficiently studied for Kazakhstan-specific raw materials, particularly in terms of their impact on reducing energy consumption in HCFeCr production.

The aim of this study is to develop a technology for producing ferrochrome using metallized chromite pellets and to investigate the influence of their preparation parameters on energy consumption and smelting efficiency.

## 2. Materials and Methods

The present study was carried out within a systematic technological framework aimed at the production of pre-reduced chromite pellets for subsequent HCFeCr smelting. The experimental methodology included several interrelated stages: characterization of the initial chromite raw materials, analysis of the carbonaceous reductant and binder, preparation of the charge mixture, pelletizing using a disk granulator, drying of the produced pellets, and their reduction roasting in a muffle furnace.

### 2.1. Chemical Composition of Raw Materials

Chromite ore from the Kempirsai deposit, located in the Aktobe region of Kazakhstan, was used in this study. The deposit belongs to the large ultramafic Kempirsai massif and is characterized by high-chromium chromite ores widely used in ferrochrome production. The ore mainly consists of chromite spinel minerals represented by magnochromite, ferrochromite, and their solid solutions, whereas the gangue material is predominantly composed of serpentine and silicate minerals [20]. The chemical composition was determined using classical wet chemical analysis methods in accordance with standard procedures. The determination of oxide components in the chromite ore was carried out according to GOST 15848.0-90 [21]. The total chromium and iron contents were determined by titrimetric methods. The chemical composition of the ore is presented in Table 1.

Semicoke was used as a carbonaceous reductant for the production of pre-reduced chromite pellets. The proximate and chemical composition of the semicoke was determined in accordance with standard methods: volatile matter according to GOST 6382-2001 [22], moisture according to GOST 27589-2020 [23]. The results are presented in Table 2.

For efficient pre-reduction, a carbonaceous reductant should be characterized by a high fixed carbon content and a low content of volatile matter. In the present study, the semicoke contains more than 80% fixed carbon and less than 7% volatile matter, indicating its suitability as a reductant for the solid-state reduction in chromite materials.

The presence of carbon in the charge ensures the reduction of iron- and chromium-bearing oxide phases during high-temperature treatment. However, the technological efficiency of the reductant is determined not only by its fixed carbon content but also by its reactivity, porosity, particle size distribution, uniformity of distribution within the charge, and interaction with the chromite phase.

Therefore, the suitability of semicoke for producing pre-reduced pellets was evaluated based on pelletizing performance, thermal treatment behavior, and the achieved degree of metallization.

Commercial sodium-activated bentonite supplied for metallurgical pelletization applications was used as a binder in this study. The bentonite was produced from natural bentonite clay subjected to industrial sodium activation in order to improve its swelling capacity, water absorption, and binding properties. The activated bentonite was used without additional laboratory treatment. Its chemical composition is presented in Table 3.

The use of activated bentonite is justified by its ability to form strong granules during pelletization of fine chromite material with a carbonaceous reductant. Upon moistening, bentonite forms a plastic binding phase that promotes adhesion between ore and semicoke particles. This is particularly important at the green pellet stage, as insufficient strength may result in pellet degradation during handling, drying, and furnace charging.

At the same time, bentonite acts as a source of additional oxides, primarily SiO_2_ and Al_2_O_3_, which can influence sintering behavior, the formation of silicate phases, and subsequent slag formation. Therefore, the bentonite content in the charge mixture must be optimized to ensure sufficient pellet strength without significantly increasing gangue content.

Thus, the selected materials—semicoke and activated bentonite—perform key technological functions: semicoke serves as a carbon source for the pre-reduction in chromite raw materials, while bentonite ensures the formation and mechanical integrity of pellets during pelletizing, drying, and subsequent high-temperature treatment.

### 2.2. Microstructural Analysis of Chromite Ore

**Figure 1:**
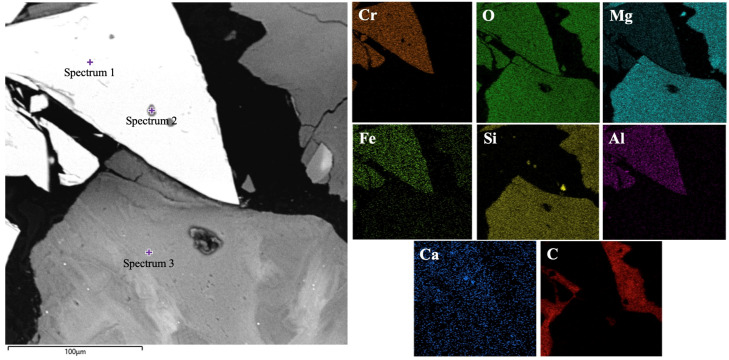
SEM micrograph and corresponding EDS elemental distribution maps of chromite ore.

The microstructural features of the initial chromite ore were investigated using scanning electron microscopy (SEM) coupled with energy-dispersive spectroscopy (EDS). The analysis was performed using a ZEM20 microscope (ZEPTOOLS, Tongling City, China) equipped with an Oxford Instruments EDS detector (Abingdon, UK). Spectra were processed using the AzTec One software package (version 6.0 SP1) with the TrueQ method. SEM images of the selected areas and corresponding elemental distribution maps are presented in Figure 1.

SEM analysis revealed that the studied material is characterized by a heterogeneous microstructure consisting of phases with different contrasts. Bright regions correspond to phases with a higher average atomic number and are associated with chromium- and iron-containing components. Gray areas are mainly attributed to magnesium- and silicon-containing phases, while dark regions correspond to pores, intergranular spaces, or areas with low signal intensity.

The results of the energy-dispersive spectroscopy (EDS) analysis of the chemical composition of selected areas are presented in Table 4.

The average elemental composition of the selected area is dominated by oxygen, magnesium, silicon, chromium, and iron. The presence of Mg, Cr, Fe, and Al indicates chromite spinel phases, while elevated Mg and Si contents are associated with silicate gangue. Significant variations in composition are observed between different spectra. Spectrum 1 corresponds to a Cr-rich spinel phase, whereas Spectrum 2 represents a mixed silicate–oxide region. Spectrum 3 is mainly composed of a magnesium silicate phase with a minor amount of iron and no detectable chromium. Overall, the ore exhibits a heterogeneous structure composed of spinel and silicate phases, which should be considered during pelletization and reduction processes.

### 2.3. Preparation of Charge Mixture and Pelletization

The charge mixture was prepared using three main components: chromite ore, semicoke, and activated bentonite. The composition of the charge mixture was selected to ensure effective pelletization and subsequent reduction roasting. The mixture consisted of 80.7 wt.% chromite ore, 16.3 wt.% semicoke, and 3.0 wt.% activated bentonite. The charge composition was calculated based on the stoichiometric carbon requirement for the reduction of chromium and iron oxides, as well as the technological requirements of pelletization and subsequent reduction roasting. The semicoke content was selected to provide sufficient fixed carbon for the reduction reactions, while the addition of activated bentonite ensured adequate mechanical strength of the green and dried pellets without significantly increasing the amount of slag-forming components. Dry mixing was carried out in a laboratory mixer for 5–7 min to obtain a homogeneous mixture. Subsequently, water was gradually added in an amount of 8–10 wt.% of the total mass. The moistened mixture was further mixed in the laboratory mixer for 2–3 min to ensure uniform moisture distribution and to obtain a plastic mass suitable for pelletization.

To break agglomerates and improve homogeneity, the wet mixture was sieved through a 1 mm mesh. The material was then conditioned for 30 min to stabilize moisture distribution, which improved binder interaction and pellet formation.

Pelletization was performed using a laboratory disk granulator. Initially, approximately 20% of the prepared mixture was loaded to form pellet nuclei, with nucleation carried out for 2 min. The remaining mixture was then added in portions, allowing pellet growth through layer-by-layer accretion.

During granulation, additional water was sprayed when necessary to maintain optimal moisture content and improve formability. After complete feeding of the mixture, the pellets were further rolled in the granulator for 2 min to enhance their mechanical strength.

### 2.4. Reduction Roasting of Chromite Pellets in a Muffle Furnace

Chromite pellets obtained after pelletization and drying were subjected to reduction roasting in a laboratory muffle furnace (Nabertherm, Lilienthal, Germany) equipped with electric heating and an automatic temperature control system. The furnace provides uniform temperature distribution within the working chamber and allows heat treatment up to 1400–1600 °C. The heating regime was controlled using a programmable controller, ensuring precise temperature regulation and holding time.

Prior to roasting, pellets without visible cracks or defects were selected. The samples were placed in refractory containers and introduced into the furnace. Roasting was carried out at 1400 °C with holding times of 1, 2, and 3 h.

After completion of the heat treatment, the samples were cooled and removed from the furnace. The roasted pellets were evaluated in terms of visual appearance, cracking, and signs of sintering. Subsequently, the samples were subjected to SEM–EDS analysis and determination of the degree of metallization.

Pre-reduction in chromite pellets at 1400 °C proceeded through the direct interaction of oxide phases with solid carbon, as well as through gas-phase reduction by carbon monoxide. The reduction degree depended on the holding time, semicoke distribution within the pellet, structural porosity, and the contact between the chromite phase and the reducing agent.

The chromium metallization degree was determined as the ratio of metallic chromium content to the total chromium content in the roasted material:

$$\alpha_{Cr}=\frac{{Cr}_{metal}}{{Cr}_{total}}\times 100\%$$

where $\alpha_{Cr}$ is the chromium metallization degree, %; ${Cr}_{metal}$ is the metallic chromium content, %; and ${Cr}_{total}$ is the total chromium content, %.

### 2.5. Smelting of Chromite Pellets in an Ore-Thermal Furnace

Technological studies on the production of HCFeCr from pre-reduced chromium pellets were carried out in a single-phase ore-thermal arc furnace equipped with a 0.1 MVA transformer and a graphite electrode 100 mm in diameter [24]. The furnace bath had dimensions of 50 × 50 cm and a depth of 35–40 cm. The furnace lining was made of magnesite and fireclay refractory bricks, while the hearth consisted of a rammed refractory mass coked for 8 h under electric current. The furnace operated in a closed mode and was equipped with a single taphole for simultaneous tapping of metal and slag.

The transformer operated at secondary voltages of 18–24 V, with a high-voltage side current of 80–100 A at a line voltage of 380 V. The arc zone temperature reached approximately 4500 °C. Prior to smelting, the furnace was preheated on a coke bed for 2 h at 24 V and 70–90 A, after which the remaining coke was removed.

Smelting was conducted continuously by feeding the charge in small portions as the burden level decreased. Metal and slag were tapped every 2 h, weighed, and sampled for chemical analysis.

## 3. Results and Discussion

### 3.1. Pelletization Results and Compressive Strength of Pellets

**Figure 2:**
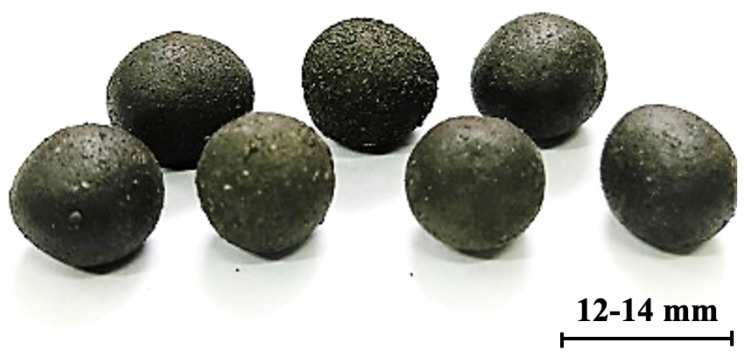
Appearance of chromite pellets (size 12–14 mm).

The produced green pellets were screened using standard sieves into the following size fractions: >14 mm, −14 + 12 mm, −12 + 10 mm, −10 + 8 mm, and <8 mm. The −14 + 12 mm fraction was selected as the target size and used for subsequent drying and reduction roasting. The undersized and insufficiently formed pellets were recycled back to the pelletization stage. The appearance of the green pellets is shown in Figure 2.

The mechanical strength of chromite pellets was evaluated to assess their suitability for drying, reduction roasting, and handling [25]. Drop strength of green pellets was determined by repeatedly dropping individual pellets from a height of 300 mm onto a steel surface until cracking or failure occurred.

The number of drops sustained was recorded. Compressive strength was measured on individual pellets by recording the maximum load at failure. Tests were conducted for green pellets, as well as after 1 and 3 days of curing. Additionally, strength after drying at 120 °C for 3 h was determined. For each condition, pellets of the same size fraction were tested, and the average compressive strength was calculated (kg/pellet). The results are presented in Table 5.

At a bentonite content of 3 wt.% in the dry charge, the produced pellets exhibited satisfactory green strength. The drop strength was at least 10 drops from a height of 300 mm before the appearance of cracks, indicating sufficient resistance to mechanical impacts during handling and preparation for drying.

The compressive strength of green pellets was 2.07 kg/pellet and increased to 4.35 and 9.70 kg/pellet after 1 and 3 days of curing, respectively. This trend indicates progressive strengthening of the pellet structure due to moisture redistribution and the development of the binding properties of bentonite.

After drying at 120 °C, the compressive strength reached 24.7 kg/pellet. The increase in strength is attributed to moisture removal and structural densification. These results confirm that the pellets possess sufficient mechanical stability for subsequent reduction roasting.

### 3.2. Reduction Roasting and SEM–EDS Analysis of Chromite Pellets

**Figure 3:**
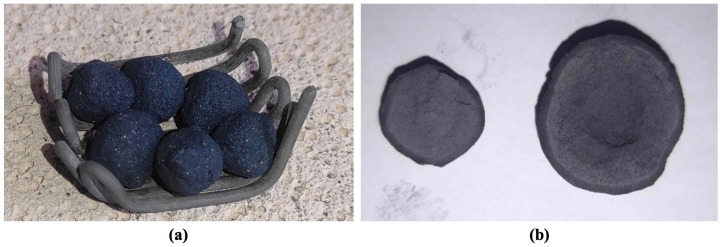
Chromite pellets after reduction roasting at 1400 °C: (**a**) external appearance; (**b**) internal structure of pellets.

Figure 3 shows chromite pellets after reduction roasting at 1400 °C. The samples exhibit a dark gray to nearly black color, which may be associated with the presence of residual carbon and reduction products.

The pellets retain their rounded shape and structural integrity without significant cracking, indicating sufficient mechanical and thermal stability during roasting. The observed heterogeneity in color, including a darker peripheral zone, may be attributed to non-uniform reduction, local sintering, and variations in reductant distribution within the pellets.

The chromium metallization degree after pre-reduction roasting at 1400 °C with holding times of 1, 2, and 3 h was determined based on the results of chemical phase analysis. The metallic chromium content was determined by selective dissolution of the metallic phase in an acid solution with minimal dissolution of chromium oxide. After separation of the insoluble residue, the chromium content in the solution was determined by titrimetric analysis. The total chromium content in the samples was determined separately by chemical analysis [26]. The metallization degree was calculated as the ratio of metallic chromium content to the total chromium content in the sample. The obtained results are presented in Table 6.

**Figure 4:**
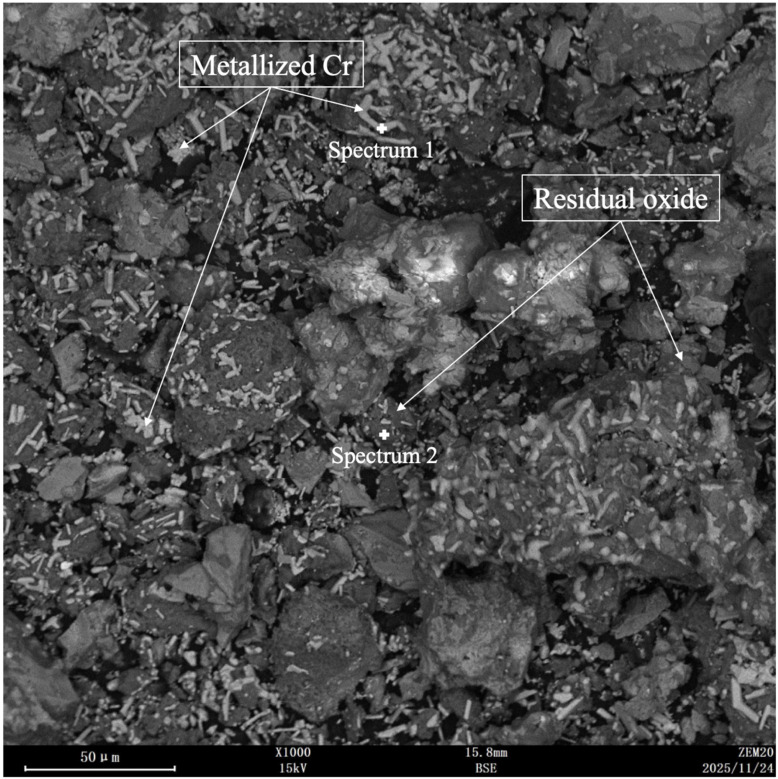
SEM image of the chromite pellet after 3 h of reduction roasting at 1400 °C.

To analyze phase transformations occurring during reduction roasting, the microstructure of the pellet after 3 h of roasting was examined using scanning electron microscopy. The corresponding SEM image is shown in Figure 4.

The SEM image reveals a heterogeneous structure with distinct phase contrast. Bright regions observed in BSE mode correspond to metallized chromium-containing phases formed during the reduction in chromite spinel.

Gray regions represent residual oxide phases that are not fully reduced, while dark areas correspond to pores and carbon-containing regions. The formation of metallized chromium-containing phases combined with developed porosity confirms the effective progression of the pre-reduction process.

Additional EDS analysis was performed to confirm the phase identification of the “Metallized Cr” region. The obtained results are presented in Table 7.

The EDS results presented in Table 7 showed that Spectrum 1 is characterized by high chromium and iron contents, confirming the formation of a metallized chromium-containing phase. Spectrum 2 is mainly composed of magnesium and aluminum oxides.

### 3.3. Smelting Performance and Energy Efficiency of Chromite Pellets

Smelting experiments were carried out using pre-reduced chromite pellets, coke, and quartzite. The charge composition was selected based on the calculated smelting conditions for HCFeCr production and consisted of 84.0% pre-reduced chromite pellets, 0.6% coke, and 15.4% quartzite.

**Figure 5:**
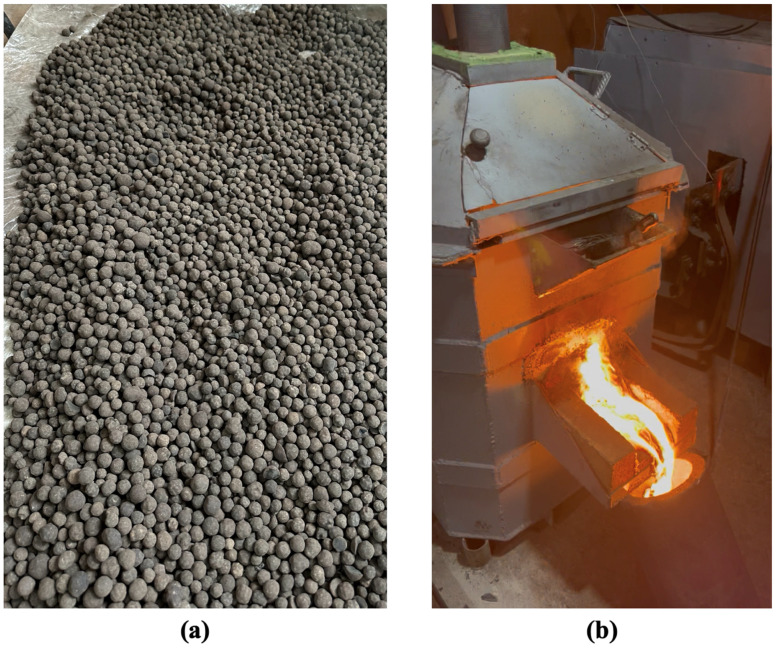
(**a**) General view of pre-reduced pellets used for smelting; (**b**) tapping process of the ferrochrome melt from the ore-thermal furnace.

The smelting process in the ore-thermal furnace and the prepared pre-reduced pellets used for ferrochrome production are shown in Figure 5.

Smelting of pre-reduced chromite pellets in the ore-thermal furnace proceeded stably without disturbances in the electrical operating regime. Uniform burden descent, stable arc operation, and satisfactory slag fluidity were observed during the smelting process. Tapping of metal and slag was carried out without difficulties, ensuring effective separation of metallic and slag phases.

**Figure 6:**
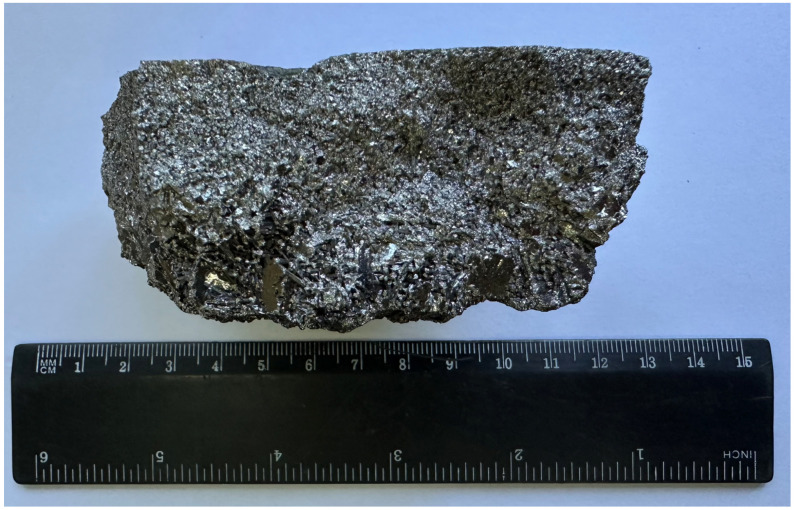
HCFeCr alloy produced from pre-reduced chromite pellets.

Six smelting experiments were carried out under identical ore-thermal furnace conditions. The produced HCFeCr obtained from smelting of the pre-reduced pellets is shown in Figure 6.

Based on the experimental results, the average composition of the obtained HCFeCr was determined as follows (wt.%): 68.92 Cr, 1.54 Si, 7.11 C, 0.015 S, and 0.040 P. The average slag composition was (wt.%): 2.14 Cr_2_O_3_, 30.12 SiO_2_, 44.32 MgO, 18.33 Al_2_O_3_, and 0.67 FeO. The chemical composition of the produced alloy corresponds to the standard requirements for HCFeCr according to GOST-4757-91 [27]. The chromium recovery into the alloy reached 92.17%. The obtained results indicate effective reduction of chromium oxides and stable separation of metal and slag during smelting of pre-reduced chromite pellets.

According to the literature data, the specific electric energy consumption for conventional HCFeCr smelting in industrial ore-thermal furnaces ranges from 3200 to 4800 kWh/t of alloy [28]. Under the conditions of smelting shop No. 4 of the Aktobe Ferroalloy Plant, this value is approximately 4800 kWh/t of ferrochrome [29].

Based on the results of smelting experiments carried out in a 0.1 MVA experimental submerged arc furnace, the specific electric energy consumption was 4648.1 kWh/t of ferrochrome. The estimation of specific electric energy consumption under industrial conditions of smelting shop No. 4 at the Aktobe Ferroalloy Plant was performed using a comparative engineering approach considering the differences in thermal efficiency, relative heat losses, furnace capacity, and operating characteristics between laboratory-scale and industrial large-capacity submerged arc furnaces. Due to the higher thermal efficiency and lower specific heat losses of industrial furnaces, the expected specific electric energy consumption under industrial conditions was estimated to be 3132.76 kWh/t of ferrochrome. The obtained value should be considered as an estimated industrial prediction rather than a direct proportional scaling result.

The obtained results demonstrate that the use of pre-reduced chromite pellets intensifies reduction processes during HCFeCr smelting and decreases specific electric energy consumption. High chromium recovery into the alloy, stable furnace operation, and low Cr_2_O_3_ content in the slag confirm the efficiency of using pre-reduced raw materials in the ore-thermal process. The expected reduction in energy consumption under industrial conditions indicates the potential applicability of this technology for ferrochrome production.

## 4. Conclusions

Pre-reduced chromite pellets for HCFeCr production were successfully produced using chromite ore, semicoke, and activated bentonite. The developed pellet composition provided satisfactory pelletizing performance and sufficient mechanical strength for subsequent thermal treatment and smelting.

Reduction roasting at 1400 °C promoted the formation of metallized chromium-containing phases. Increasing the holding time from 1 to 3 h significantly increased the chromium metallization degree, reaching 43.93% after 3 h of roasting. SEM analysis confirmed the formation of metallized regions and the development of a porous structure favorable for further reduction during smelting.

Smelting of pre-reduced pellets in a 0.1 MVA ore-thermal furnace proceeded under stable electrical and technological conditions, with satisfactory slag fluidity and effective separation of metal and slag phases. The obtained HCFeCr contained 68.92 wt.% Cr and was characterized by low sulfur and phosphorus contents. Chromium recovery into the alloy reached 92.17%, while the slag contained only 2.14 wt.% Cr_2_O_3_.

The specific electric energy consumption during experimental smelting was 4648.1 kWh/t of ferrochrome. Recalculation to industrial conditions of the Aktobe Ferroalloy Plant indicated an expected energy consumption of 3132.76 kWh/t, demonstrating the potential for reducing energy consumption compared with conventional smelting technology.

The obtained results confirm the technological efficiency and industrial potential of using pre-reduced chromite pellets for the energy-efficient production of HCFeCr.

## Figures and Tables

**Table 1:** Chemical composition of chromite ore (wt.%).

Cr_2_O_3_	FeO	Al_2_O_3_	MgO	SiO_2_	CaO	Moisture	Cr_total_	Fe_total_	Cr/Fe
47.10	12.25	6.94	20.92	9.00	0.29	10.70	32.23	9.50	3.38

**Table 2:** Proximate and chemical composition of semicoke (wt.%).

Moisture	C_fixed_	VM	FeO	Al_2_O_3_	MgO	SiO_2_	CaO	P	S
19.9	87.5	5.76	0.43	1.50	0.12	3.94	0.18	0.015	0.092

**Table 3:** Chemical composition of activated bentonite (wt.%).

SiO_2_	MgO	Al_2_O_3_	CaO	FeO	Moisture	Other
59.30	3.80	15.10	0.60	4.10	9.63	10.70

**Table 4:** EDS analysis of selected areas of chromite ore (weight %).

Element	Average Composition	Spectrum 1	Spectrum 2	Spectrum 3
Cr	11.33	27.30	6.68	—
O	54.71	48.84	56.35	58.95
Mg	15.99	11.96	11.95	24.06
Fe	2.70	5.90	1.50	0.70
Si	10.10	—	14.12	16.19
Al	3.53	6.00	4.60	—
Ca	1.60	—	4.81	—
Total	100.00	100.00	100.00	100.00

**Table 5:** Mechanical strength of chromite pellets.

Drop Strength, Number of Drops	Compressive Strength, kg/Pellet	After 1 Day	After 3 Days	After Drying at 120 °C
≥10	2.07	4.35	9.70	24.7

**Table 6:** Chemical composition of the obtained pellets and chromium metallization degree (wt.%).

Holding Time, h	Cr_2_O_3_	FeO	Al_2_O_3_	MgO	SiO_2_	CaO	C	Cr_metal_	Degree of Metallization (%)
1	45.86	12.15	6.90	20.91	8.89	0.26	5.03	-	-
2	42.04	12.01	6.87	20.67	8.78	0.28	5.89	3.46	10.74
3	25.07	11.89	6.53	20.45	8.55	0.29	12.76	14.46	43.93

**Table 7:** EDS point analysis of the chromite pellet (weight %).

Element	Spectrum 1	Spectrum 2
C	1.24	8.65
O	1.84	47.20
Mg	0.94	25.78
Al	0.47	13.45
Si	0.12	1.66
Cr	84.98	2.14
Fe	10.41	1.12
Total	100.00	100.00

## Data Availability

The original contributions presented in this study are included in the article. Further inquiries can be directed to the corresponding authors.

## References

[1] Basson J., Daavittila J. (2013). High Carbon Ferrochrome Technology. Handbook of Ferroalloys: Theory and Technology.

[2] Gasik M.I. (2013). Technology of Chromium and Its Ferroalloys. Handbook of Ferroalloys: Theory and Technology.

[3] Downing J.H., Deeley P.D., Fichte R. (2000). Chromium and Chromium Alloys. Ullmann’s Encyclopedia of Industrial Chemistry.

[4] Sariyev O., Almagambetov M., Nurgali N., Bilyalov K., Kelamanov B., Yessengaliyev D., Abdirashit A. (2026). Briquetting and Remelting of Aspiration Dust Generated During High-Carbon Ferrochrome Crushing in Direct Current Electric Arc Furnaces. Materials.

[5] Ringdalen E., Rocha M., Figueiredo P. Energy consumption during HCFeCr-production at Ferbasa. Proceedings of the Fourteenth International Ferroalloys Congress.

[6] Sariyev O., Almagambetov M., Nurgali N., Abikenova G., Kelamanov B., Yessengaliyev D., Abdirashit A. (2025). Development of a Briquetting Method for Dust from High-Carbon Ferrochrome (HC FeCr) Crushing Using Vibropressing on an Industrial Scale and Its Subsequent Remelting. Materials.

[7] International Chromium Development Association (ICDA) Market Insights (Chromite and Ferrochrome Statistics). https://www.icdacr.com/market-insights/.

[8] Dehghanpour H., Doğan F., Subaşı S., Maraşlı M. (2022). Effects of single-walled carbon nanotubes and steel fiber on recycled ferro-hrome filled electrical conductive mortars. J. Sustain. Constr. Mater. Technol..

[9] du Preez S.P., van Kaam T.P.M., Ringdalen E., Tangstad M., Morita K., Bessarabov D.G., van Zyl P.G., Beukes J.P. (2023). An Overview of Currently Applied Ferrochrome Production Processes and Their Waste Management Practices. Minerals.

[10] Panda C.R., Mishra K.K., Nayak B.D., Rao D.S., Nayak B.B. (2012). Release behaviour of chromium from ferrochrome slag. Int. J. Environ. Technol. Manag..

[11] Kleynhans E.L.J., Beukes J.P., Van Zyl P.G., Bunt J.R., Nkosi N.S.B., Venter M. (2017). The Effect of Carbonaceous Reductant Selection on Chromite Pre-Reduction. Metall. Mater. Trans. B.

[12] McCullough S., Hockaday S., Johnson C., Barcza N.A. Pre-reduction and smelting characteristics of Kazakhstan ore samples. Proceedings of the INFACON XII.

[13] Glastonbury R.I., Beukes J., Van Zyl P., Sadikit L., Jordaan A., Campbell Q., Stewart H., Dawson N. (2015). Comparison of physical properties of oxidative sintered pellets produced with UG2 or metallurgical-grade South African chromite: A case study. J. S. Afr. Inst. Min. Metall..

[14] Kleynhans E., Neizel B., Beukes J., Van Zyl P. (2016). Utilisation of pre-oxidised ore in the pelletised chromite pre-reduction process. Miner. Eng..

[15] Xing J., Zhang J., Wang Y., Luo G., An S., Jin Y., Li Y., Chai Y. (2026). Hydrogen-based rotary kiln reduction of magnetite-based oxidized pellets: A comprehensive study from macro-performance to micro-mechanisms. Int. J. Hydrogen Energy.

[16] Shotanov A.E., Roshchin A.V., Panfilov V.P., Nurgali N.Z. (2022). Prereduction of Chromite Raw Materials by the Höganäs Method. Metallurgist.

[17] Shotanov A.E., Nurgali N.Z., Roshchin A.V., Panfilov V.P., Baysanov S.O., Almagambetov M.S. (2023). Smelting of High-Carbon Ferrochrome from Prereduced Chromite Raw Materials of the Donskoy Ore Mining and Processing Plant. Metallurgist.

[18] Baysanov S., Shabanov Y.Z., Grigorovich K.V., Toleukadyr R.T., Inkarbekova I.S. (2022). Smelting options for carbon ferrochrome based on ore raw materials, middlings and their technological evaluation. Kompleks. Ispolz. Miner. Syra.

[19] Kumar P., Patra S.K., Tripathy S.K., Sahu N. (2021). Efficient utilization of nickel rich Chromite Ore Processing Tailings by carbothermic smelting. J. Clean. Prod..

[20] Orlov A.S. (2020). Research and Development of Aluminum–Chromium–Silicon Alloy Smelting Technology Using High-Ash Borlinsk Coal as a Reducing Agent. Ph.D. Thesis.

[21] (1992). Chromite Ores and Concentrates. General Requirements for Methods of Chemical and Physicochemical Analysis.

[22] (2003). Hard Coal and Coke. Determination of Volatile Matter.

[23] (2020). Coke. Determination of Moisture in the Analysis Sample.

[24] Makhambetov Y., Zhakan A., Zhunusov A., Kabylkanov S., Burumbayev A., Sadyk Z., Akhmetov A., Uakhitova B. (2025). Resource-Efficient Smelting Technology for FeCrMnSi Ferroalloy Production from Technogenic Wastes in an Ore-Thermal Furnace. Metals.

[25] (2015). Iron Ore Pellets—Determination of Crushing Strength.

[26] (1985). Method for Determination of Chromium.

[27] (1991). Ferrochrome—Technical Requirements and Delivery Conditions.

[28] Aristotel I., Zhalgas S., Saule S., Yerbolat M. (2026). Ferrochrome Smelting Using Chrome Raw Materials Pre-Reduced with Various Reducing Agents. Metals.

[29] Shabanov Y., Makhambetov Y., Saulebek Z., Toleukadyr R., Baisanov S., Nurgali N., Shotanov A., Dossekenov M., Zhumagaliyev Y. (2024). Pilot Tests of Pre-Reduction in Chromium Raw Materials from Donskoy Ore Mining and Processing Plant and Melting of High-Carbon Ferrochromium. Metals.

